# 

**Published:** 1885-12

**Authors:** 


### CONTENTS.

Original Articles—Remarks on Spinal Abscesses—Revelations by Electricity—Case of Peri-Uterine Cellulitis ........ 249–261
Cullings from Contemporaries—A few Wholesome but Unpalatable Facts—Another One — How to Build up a Medical Society—Successful Cæsarian Section After Death of Mother ........ 263–266
Correspondence—Carbolic Acid in Carbuncle Our New York Letter, Bone Surgery ........ 266–267
Society Notes—East Line Medical Association—Our Transactions—Seed Sown by the Wayside ... 270–271
Editorial — Why Physicians Cannot Consult with Homœopaths—First Death in P. M. B. A.—Wants to Know You Know—A Two Edged Sword—A New and Valuable Diagnostic System—Overtures to Peace—Yellow Fever in Galveston—More Piracy—Gaillard's Medical Journal—Bravo—Vice-President Hendrick's Disease — Our Agents — Blue's Elegy ........ 275–289
Necrology—Dr. W. H. Park ........ 290
Bibliography—Books Received, Reviews ........ 291
Melange ........ 294
Advertisers' Editorials ........ 295

